# Central Nervous System Metabolism in Autism, Epilepsy and Developmental Delays: A Cerebrospinal Fluid Analysis

**DOI:** 10.3390/metabo12050371

**Published:** 2022-04-20

**Authors:** Danielle Brister, Brianna A. Werner, Geoffrey Gideon, Patrick J. McCarty, Alison Lane, Brian T. Burrows, Sallie McLees, P. David Adelson, Jorge I. Arango, William Marsh, Angelea Flores, Matthew T. Pankratz, Ngoc Han Ly, Madison Flood, Danni Brown, David Carpentieri, Yan Jin, Haiwei Gu, Richard E. Frye

**Affiliations:** 1 Barrett, The Honors College, Arizona State University, Tempe, AZ 85281, USA; dbriste1@asu.edu; 2 Section on Neurodevelopmental Disorders, Barrow Neurological Institute at Phoenix Children’s Hospital, Phoenix, AZ 85016, USA; bwerner@phoenixchildrens.com (B.A.W.); pmccarty@phoenixchildrens.com (P.J.M.); alane@phoenixchildrens.com (A.L.); smclees@phoenixchildrens.com (S.M.); maddieflood@gmail.com (M.F.); 3 Department of Child Health, University of Arizona College of Medicine-Phoenix, Phoenix, AZ 85004, USA; 4 Arizona College of Osteopathic Medicine, Midwestern University, Glendale, AZ 85308, USA; ggideon61@midwestern.edu; 5 Division of Neuroscience, Barrow Neurological Institute at Phoenix Children’s Hospital, Phoenix, AZ 85016, USA; bburrows@phoenixchildrens.com (B.T.B.); dadelson@phoenixchildrens.com (P.D.A.); jarango@phoenixchildrens.com (J.I.A.); hly@phoenixchildrens.com (N.H.L.); dbrown4@phoenixchildrens.com (D.B.); 6 Mayo Clinic, Scottdale, AZ 85259, USA; marsh.william@mayo.edu; 7 Department of Pathology, Phoenix Children’s Hospital, Phoenix, AZ 85016, USA; aflores@phoenixchildrens.com (A.F.); mpankratz@phoenixchildrens.com (M.T.P.); dcarpentieri@phoenixchildrens.com (D.C.); 8 Center for Translational Science, Florida International University, Port St. Lucie, FL 34987, USA; yajin@fiu.edu (Y.J.); hgu@fiu.edu (H.G.)

**Keywords:** amino acid metabolism, autism spectrum disorder, cerebrospinal fluid, *cis-cis*-muconic acid, developmental delay, energy metabolism, epilepsy, mass spectrometry, metabolomics, redox metabolism, shikimic acid, vitamins

## Abstract

Neurodevelopmental disorders are associated with metabolic pathway imbalances; however, most metabolic measurements are made peripherally, leaving central metabolic disturbances under-investigated. Cerebrospinal fluid obtained intraoperatively from children with autism spectrum disorder (ASD, *n* = 34), developmental delays (DD, *n* = 20), and those without known DD/ASD (*n* = 34) was analyzed using large-scale targeted mass spectrometry. Eighteen also had epilepsy (EPI). Metabolites significantly related to ASD, DD and EPI were identified by linear models and entered into metabolite–metabolite network pathway analysis. Common disrupted pathways were analyzed for each group of interest. Central metabolites most involved in metabolic pathways were L-cysteine, adenine, and dodecanoic acid for ASD; nicotinamide adenine dinucleotide phosphate, L-aspartic acid, and glycine for EPI; and adenosine triphosphate, L-glutamine, ornithine, L-arginine, L-lysine, citrulline, and L-homoserine for DD. Amino acid and energy metabolism pathways were most disrupted in all disorders, but the source of the disruption was different for each disorder. Disruption in vitamin and one-carbon metabolism was associated with DD and EPI, lipid pathway disruption was associated with EPI and redox metabolism disruption was related to ASD. Two microbiome metabolites were also detected in the CSF: shikimic and *cis-cis*-muconic acid. Overall, this study provides increased insight into unique metabolic disruptions in distinct but overlapping neurodevelopmental disorders.

## 1. Introduction

Neurodevelopmental disorders have increased in prevalence over in recent decades. For example, the most recent Center for Disease Control and Prevention estimation is that autism spectrum disorder (ASD) affects approximately 2% of children in the United States, with the prevalence continuing to increase [1]. Developmental delay (DD) and epilepsy (EPI) continue to increase in prevalence. ASD [2] and EPI [3] overlap with psychiatric disorders, which are also growing in prevalence in children and adolescents [4]. 

Although some individuals with these neurodevelopmental issues have identifiable genetic etiologies, treatment is still limited. In many, the genetic underpinnings remain elusive. Thus, a better understanding of the underlying physiological processes could provide insight into novel treatment targets.

Abnormalities in metabolic processes are linked to neurologic and neurodevelopmental disorders. For example, converging lines of evidence suggest that mitochondrial dysfunction and oxidative stress are common factors in many neurodevelopmental disorders, including ASD [5,6], Rett syndrome [7] and Down syndrome [8]. Along with the immune system, these pathophysiological processes are described as the ‘Bad Trio’ that may lead to neurodevelopmental disease [9]. Of course, other important metabolic systems play a role in brain function, particularly amino acid and non-amino acid neurotransmitters. Furthermore, deficiency and insufficiency of specific vitamins have been related to neurodevelopmental disorders. For example, disruption in central folate availability is associated with EPI, DD and ASD and central pyridoxine deficiency is a rare but well-known cause of EPI in neonates and infants. 

Several metabolomics studies have been conducted on children with neurodevelopmental disorders (Table 1). 

Children with DD have been shown to have increased glycolic and 3-hydroxyisobutyric acid and decreased palmitic acid [21] in urine and elevated acetate, glutamate, lactate and tricarboxylic acid (TCA) cycle metabolites in plasma [13]. Interestingly, one study that included DD and ASD participants demonstrated that cognitive function, adaptive skills, and aberrant behavior were related to some of these same metabolites [14]. 

Metabolomics studies on individuals with EPI have looked at blood, cerebrospinal fluid (CSF) and post-mortem brain (Table 1). Studies on blood have identified changes in amino acid neurotransmitter metabolism with a consistent finding of increased glutamate [10,11,12]. Changes in fatty acid and energy metabolism have also been consistently found in blood, but other metabolites varied from study to study [10,11,12]. One study on CSF in EPI found changes in metabolites associated with altered pyridoxine metabolism in preschool children and a reduction in 1,5-anhydroglucitol in school-aged children [22]. One study on post-mortem frontal lobes in adults with idiopathic EPI found alterations in metabolites involved in fatty acid, pentose phosphate pathways, vitamin pathways including thiamine, one-carbon, nicotinamide, pantothenate and CoA pathways as well as amino acid metabolism including phenylalanine and tyrosine [24]. 

Many systemic metabolic abnormalities that can be detected by peripheral fluids such as blood and urine are known to be associated with ASD. While 5% of children with ASD exhibit mitochondrial disease [25], up to 80% may demonstrate mitochondrial dysfunction [26]. Abnormalities in standard blood biomarkers of mitochondrial dysfunction such as lactate, pyruvate, alanine, creatine kinase, ammonia and carnitine [25] as well as fatty acid oxidation biomarkers are prevalent in the ASD population [27,28]. Abnormalities in folate one-carbon metabolism (FOCM) and its downstream effects on methylation, transsulfuration, redox metabolism and oxidative stress were originally described as an ASD endophenotype [29,30] but have recently been provisionally shown to be potentially diagnostic [31,32,33]. Lastly, dysregulation of branched-chain amino acids as well as glutamine, glycine, and ornithine has been related to an ASD subgroup [34]. 

Studies on blood amino acids in ASD tend to find variable changes but, overall, studies suggest lower overall amino acid concentrations in the blood [25] and urine [19]. Several studies have examined urine metabolites (Table 1), with one recent untargeted metabolomics study demonstrating abnormalities in monoamine neurotransmitters, 4-cresol and pyridoxal-5-phosphate, suggesting metabolic changes linked to the enteric microbiome [20]. Another study demonstrated that urine metabolites of propionate were related to gastrointestinal (GI) symptoms in children with ASD [19]. Direct analysis of stool metabolites has identified alteration in acyl-carnitine and nicotinamide metabolites [15], isopropanol [16], phenol compounds [17] and indole [17,18] related to ASD (Table 1). 

Abnormalities in central metabolic processes detected by examination of the CSF are also associated with ASD. Perhaps one of the most significant metabolic abnormalities in the CSF known to affect children with ASD is central folate abnormalities [35], while other children with ASD have been shown to have deficiency in another important cofactor, tetrahydrobiopterin [36]. One study that profiled the metabolome of the posterolateral cerebellum from post-mortem individuals with ASD as compared to age-matched controls found disruption in pyrimidine, ubiquinone and vitamin K metabolism along with elevations in long-chain fatty acids [23]. 

While studies have suggested that ASD might be associated with unique central metabolic abnormalities, such as amino acid availability [37], few studies have directly measured metabolite differences in CSF of children with ASD. Understanding what metabolic changes in the brain are specific to which neurodevelopmental disorders can improve our understanding of disease and potential treatments. The study of CSF is essential as the blood–brain barrier highly regulates the movement of metabolites between the blood and brain [38]. Thus, metabolic changes in the brain are difficult to measure accurately in the blood, limiting our understanding regarding whether metabolic changes found using peripheral biomarkers in blood, urine or stool are also acting in the brain. Thus, in this study, we used targeted metabolomics to identify metabolic changes in the brain associated with EPI, DD and ASD by examining CSF obtained intraoperatively. These clinical groups were compared to control CSF samples of patient without these neurodevelopmental conditions. Using linear modeling, the analysis in this paper is able to identify changes in each clinical group while controlling for the effects of the other clinical groups. Eighty eight samples were matched for important characteristics including sampling locations. From review of the literature, we find that this is largest study to date on CSF in neurological and/or neurodevelopmental disorders in childhood. 

## 2. Results

Several analysis methods were used to analyze CSF metabolites. After normalization, linear regression models determined which metabolites were significantly altered in each neurodevelopmental disorder group while controlling for the others, thus controlling for potentially overlapping clinical characteristics. These metabolites were then analyzed using metabolite–metabolite interaction analysis. General themes regarding the implicated pathways for each neurodevelopmental disorder are summarized. 

### 2.1. Linear Models

**Figure 1:**
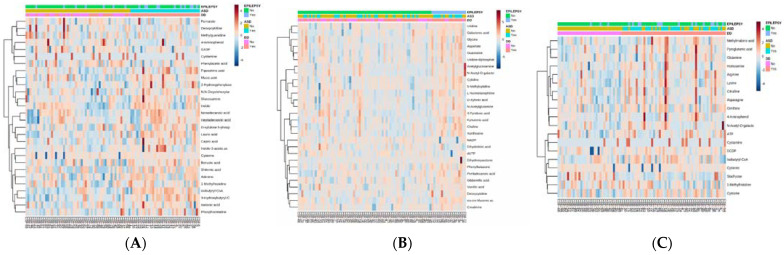
Heatmaps for Metabolites Found to Be Significantly (*p* ≤ 0.05) Related to Specific Clinical Groups. Each map is sorted by clinical group with color key for group membership on top of heatmap. Heatmap color represents (**A**) ASD (28 significant features); (**B**) epilepsy (28 significant features); (**C**) DD (19 significant features).

Figure 1 provides the heatmaps of the significant metabolites associated with each neurodevelopmental disorder group and Supplementary Table S1 provides the detailed statistics for each significant metabolite. Metabolites which were statistically significant (*p* ≤ 0.01) and demonstrated marked fold change (FC > 1.5) are outlined for each neurodevelopmental disorder group. For the ASD group, these metabolites include isobutyryl-CoA [t = 6.86, *p* < 0.0001; FC 2.1], shikimic acid [t = 3.39, *p* = 0.001; FC 2.2] and cysteine [t = 3.13, *p* < 0.01; FC 1.7]. For the EPI group, these metabolites include cytidine [t = 3.79, *p* < 0.001; FC 1.5], deoxycytidine [t = 3.14, *p* < 0.01; FC 1.5], *cis-cis*-muconic acid [t = 2.95, *p* < 0.01; FC 1.7] and guanosine [t = 2.82, *p* < 0.01; FC 1.5]. For the DD group, these metabolites include cystamine [t = 3.16, *p* < 0.01; FC 1.8] and adenosine triphosphate (ATP) [t = 2.80, *p* < 0.01; FC 1.5]. 

### 2.2. Metabolite–Metabolite Network Analysis

#### 2.2.1. Pathway Analysis

**Figure 2:**
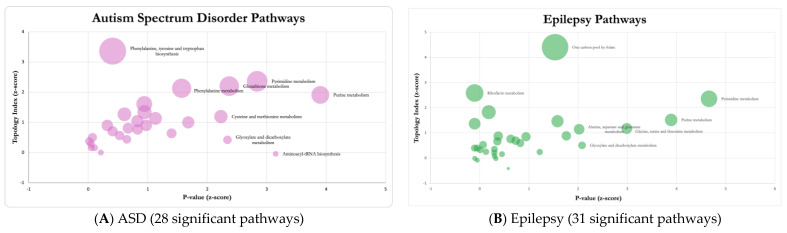

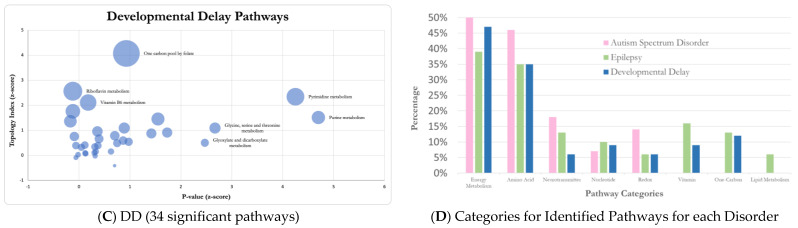
Major Nodes for Metabolite–Metabolite Interaction Networks for (**A**) Autism Spectrum Disorder (ASD), (**B**) epilepsy (EPI) and (**C**) Developmental Delay (DD) as well as (**D**) Categories for Pathways Identified for each Disorder.

Figure 2 outlines the major pathways involved in metabolite–metabolite interactions for each neurodevelopmental disorder group (Figure 2A–C) as well as their overlapping features (Figure 2D). Prominent pathways (*p* z-value or Topo z-value ≥ 2SD) for each neurodevelopmental disorder group are labeled in Figure 2A–C. 

Prominent pathways for the ASD group (Figure 2A) include purine metabolism [p_z_ = 3.90, Topo_z_ = 1.91], aminoacyl-tRNA biosynthesis [p_z_ = 3.15, Topo_z_ = −0.04], pyrimidine metabolism [p_z_ = 2.74, Topo_z_ = 2.36], glutathione metabolism [p_z_ = 2.37, Topo_z_ = 2.20], glyoxylate and dicarboxylate metabolism [p_z_ = 2.34, Topo_z_ = 0.43], cysteine and methionine metabolism [p_z_ = 2.23, Topo_z_ = 1.19] and phenylalanine metabolism [p_z_ = 1.57, Topo_z_ = 2.13]. Prominent pathways for the EPI group (Figure 2B) include pyrimidine metabolism [p_z_ = 4.66, Topo_z_ = 2.36], purine metabolism [p_z_ = 3.89, Topo_z_ = 1.52], glycine, serine and threonine metabolism [p_z_ = 2.99, Topo_z_ = 1.18], glyoxylate and dicarboxylate metabolism [p_z_ = 2.08, Topo_z_ = 0.51], alanine, aspartate and glutamate metabolism [p_z_ = 2.02, Topo_z_ = 1.15], one-carbon pool by folate [p_z_ = 1.53, Topo_z_ = 4.41] and riboflavin metabolism [p_z_ = −0.11, Topo_z_ = 2.59]. Prominent pathways for the DD group (Figure 2C) include purine metabolism [p_z_ = 4.70, Topo_z_ = 1.51], pyrimidine metabolism [p_z_ = 4.25, Topo_z_ = 2.34], glycine, serine and threonine metabolism [p_z_ = 2.67, Topo_z_ = 1.09], glyoxylate and dicarboxylate metabolism [p_z_ = 2.47, Topo_z_ = 0.51], one-carbon pool by folate [p_z_ = 0.93, Topo_z_ = 4.07] and vitamin B6 metabolism [p_z_ = 0.18, Topo_z_ = 2.11]. 

The pathways were divided into eight metabolic categories for each clinical group. The percentage of each metabolic category as a percentage of the total number of metabolic pathways identified is provided in Figure 2D. Energy and amino acid pathways predominated, representing 45% and 39% of the pathways, respectively. Neurotransmitters represented 12% of the pathways, while nucleotide and redox metabolism represented 9% of the metabolites each. Vitamin and one-carbon metabolism were only found in EPI and DD groups and made up 8% of the pathways each overall. Lipid metabolism was only found in the epilepsy group.

**Figure 3:**
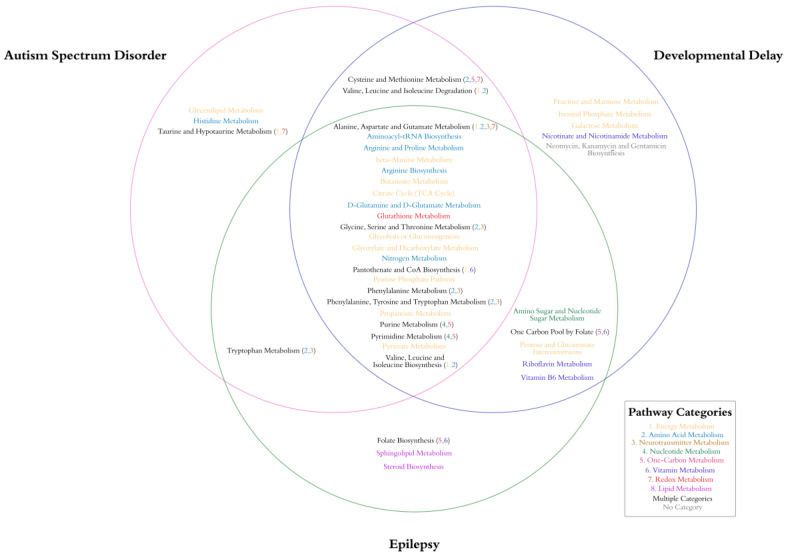
Metabolite–Metabolite Network Pathway Overlap by Clinical Group. Color code represents pathway category. Metabolites in black belong to multiple pathways which are specified by colored numbers in parenthesis following the metabolite name.

Figure 3 depicts the overlap of the metabolic pathways identified for the various neurodevelopmental groups. As can be seen in Figure 3, many pathways overlap for all three disorders, predominately energy, amino acid and neurotransmitter metabolism. Other pathways are shared by two or more disorders. Amino acids important in energy, one-carbon and redox metabolism are shared by ASD and DD, while ASD and EPI share tryptophan, an essential α-amino acid important in the production of the neurotransmitter serotonin, the hormone melatonin and the vitamin niacin. Interestingly, shared between DD and EPI are multiple vitamin pathways including folate, riboflavin and pyridoxine metabolism. DD showed exclusive relation to nicotinamide metabolism. Each disorder has exclusive association with specific pathways. ASD is exclusively related to the glycolipid, histidine and taurine/hypotaurine metabolism. EPI is exclusively related to sphingolipid and steroid metabolism, while DD is exclusively related to non-mitochondrial carbohydrate metabolism. 

#### 2.2.2. Pathway Analysis

**Figure 4:**
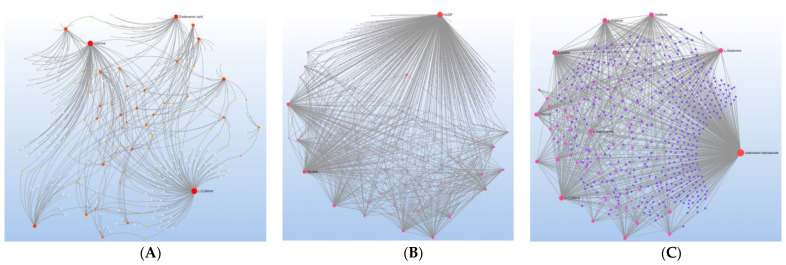
Major Nodes for Metabolite–Metabolite Interaction Networks for (**A**) ASD, (**B**) epilepsy (EPI) and (**C**) DD.

Figure 4 depicts the overall metabolite–metabolite interaction for each disorder with the major nodes highlighted to depict the major metabolic drivers of the networks. For ASD, the major nodes consist of L-cysteine, adenine and dodecanoic acid. For EPI, the major node was nicotinamide adenine dinucleotide phosphate (NADP), with glycine as a slightly smaller node. For DD, adenosine triphosphate (ATP) was the major node, with several amino acids as slightly smaller nodes, including L-glutamine, L-cysteine, citrulline L-lysine, L-arginine and ornithine.

#### 2.2.3. Interaction of Major Nodes with Other Metabolites

**Figure 5:**
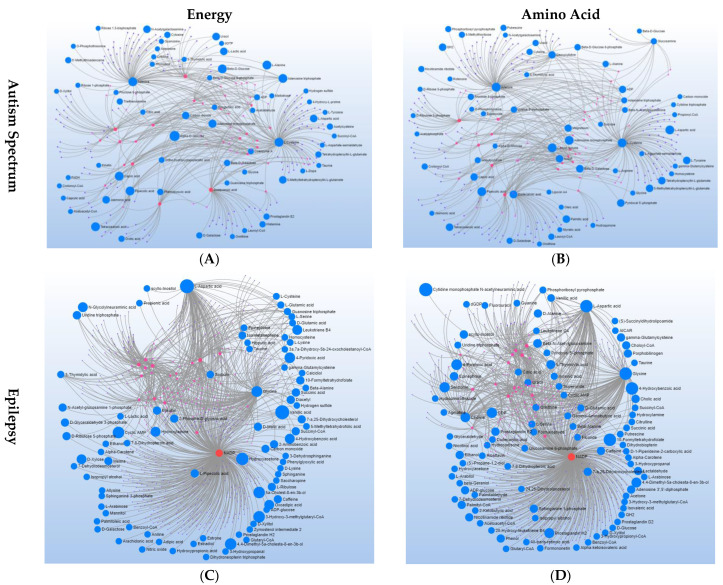

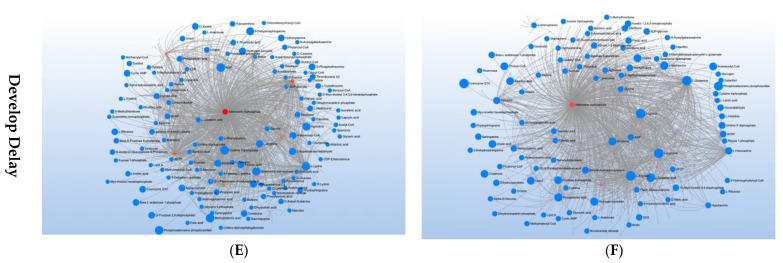
Metabolite–Metabolite Interaction Networks for Energy and Amino Acid Pathways in ASD (**A**,**B**), epilepsy (EPI) (**C**,**D**) and DD (**E**,**F**) Groups.

Interestingly, although all three neurodevelopmental disorders share abnormalities within specific pathways, the way in which they interact with metabolites in these pathways differs from disorder to disorder. For example, for the energy pathways, the metabolite–metabolite interaction networks demonstrate the unique nature of energy metabolism in each disorder. For ASD, the major nodes of the network (L-cysteine, adenine and dodecanoic acid) are all connected through ATP (Figure 5A), the major energy molecule of the mitochondria. For EPI, the metabolites connected to the major node of the network NADP are linked because NADP is the important energy supplier for the reactions that produce these metabolites (Figure 5C). For DD, the relationships of the major node, ATP, to other metabolites are through support of mitochondrial function to produce ATP (Figure 5E). 

Similar to energy pathways, the metabolite–metabolite interaction networks for amino acids demonstrate the unique nature of amino acid metabolism in each disorder. For ASD, there are three major metabolites (Figure 5B): L-cysteine is connected to either amino acid precursors (homocysteine) or transsulfuration metabolites (e.g., L-cystine and L-cystathionine) involved in the production of glutathione (GSH); adenine is associated with other purines; dodecanoic acid is related to other medium-chain fatty acids. Similar to the pattern seen for energy metabolism, for EPI, metabolite connections to NADP are linked because NADP is an important cofactor for reactions that produce the metabolites (Figure 5D). Additionally, like the pattern seen for energy metabolism, for DD, the connection of other metabolites to ATP is through support of mitochondrial function to produce ATP.

These patterns of distinct differences between disorders within various pathways and the primary contribution of major network nodes to other metabolites are similar for other pathways including neurotransmitter (Supplementary Figure S2), redox (Supplementary Figure S3), nucleotide (Supplementary Figure S4), one-carbon (Supplementary Figure S5), vitamin (Supplementary Figure S6) and lipid (Supplementary Figure S7) metabolism.

## 3. Discussion

This study examined the metabolic profiles in CSF of three neurodevelopmental disorders, ASD, EPI and DD, using statistical methods to extract differentially expressed metabolites while controlling for the potential overlap between these disorders. Overall, each disorder demonstrated some unique metabolic changes that converged on many overlapping pathways. Prominent in the metabolite–metabolite interaction networks were energy and amino acid pathways commonly involved in each disorder, but each disorder demonstrated unique interactions between the major metabolites with other metabolites in these pathways. While ASD demonstrated that L-cysteine and adenine appeared to be prominent intermediates in many related pathways, EPI pathways demonstrated the importance of NADP as an important cofactor for metabolic reactions and DD demonstrated the importance of ATP as an important component of mitochondrial function. 

For ASD, the major nodes included adenine, L-cysteine and dodecanoic acid (aka lauric acid). These metabolites are related to systems that have been previously implicated in ASD. Adenine is central in the cell danger response (CDR) hypothesis described by Dr Robert Naviaux [39,40]. The CDR is the evolutionarily conserved metabolic response that has been hypothesized to protect cells in the face of external (e.g., environmental) threats. Disease is proposed to occur when the CDR does not resolve and continues chronically. CDR explains some of the key metabolic abnormalities associated with ASD and treatment that interrupts the CDR demonstrates improvement in core and associated ASD behaviors [39,40]. L-cysteine is a major component of transsulfuration. As previously pointed out, ASD is associated with alternations in FOCM and its resulting secondary effects, including those on methylation, transsulfuration and redox metabolism, as well as oxidative stress [29,30]. Originally, Dr. S. Jill James described this as a unique ASD endophenotype [29] in patients with ASD. However, these abnormalities affect most patients and may be diagnostic of ASD. For example, using Fisher discriminant analysis, individuals with ASD can be differentiated from TD children using these FOCM and related metabolites with an 88% to 97% accuracy [31,32,33]. Dodecanoic acid (aka lauric acid) is an interesting medium-chain saturated fatty acid which is the major component of coconut oil. Interestingly, it is not uncommon to see carnitine deficiency in ASD [28] and lauric acid builds up when the carnitine shuttle is deficient [41]. 

EPI was associated with changes in NADP metabolism. NADP is connected to other metabolic reactions through itself and its redox couple (NADPH) as essential cofactors in numerous important cellular pathways [42]. NADP is important in TCA intermediate production (isocitrate dehydrogenase), pyruvate metabolism (malate dehydrogenase), mitochondrial proton-translocation (nicotinamide nucleotide transhydrogenase), both cytosolic (MTHFD1) and mitochondrial-specific (MTHFD2, MTHFD1L, MTHFD2L, ALDH1L2) folate metabolism and glutamate deamination (glutamate dehydrogenase), while NADPH is essential for fatty acid synthesis, steroidogenesis, drug metabolism and heme degradation (3-hydroxy-3-methylglutaryl-CoA reductase, NADPH-cytochrome P450 oxidoreductase) [42,43] as well as ubiquinol production (NADPH-CoQ reductase) [44] and as a cofactor for thioredoxin reductases, which are integral to redox and nitric oxide metabolism and DNA and protein repair [45]. The recently described mitochondrial localized NAD kinase MNADK is essential for NADP biosynthesis in the mitochondrial compartment independent of the cytosol and plays a pivotal role in mitochondrial function, particularly with respect to redox regulation [46]. Loss of this enzyme results in a mitochondrial disease phenotype emphasizing the importance of NADP in mitochondrial function [46]. In addition, EPI was the only disorder associated with changes in lipid metabolism, consistent with previous studies [10,11,12,24]. This can be directly linked back to NADP/NADPH metabolism [42,43]. 

EPI was associated with abnormalities in several vitamin pathways, including folate, pyridoxine, riboflavin and nicotinamide. Studies on children with EPI have identified abnormalities in pyridoxine metabolism in CSF [22] and pyridoxine and nicotinamide metabolism in post-mortem brain [24]. Interesting studies in post-mortem brain have identified abnormalities in other vitamin pathways including thiamine as well as pantothenate and its important product for energy metabolism CoA [24]. Consistent with our findings, EPI is associated with several vitamin deficiencies. Pyridoxine-dependent and -responsive epilepsy are well known but perhaps underappreciated. Typically associated with neonatal seizures, pyridoxine-dependent epilepsy is also described in adolescents [47] and associated with metabolic disturbances such as oxidative stress [48]. Cerebral folate deficiency (CFD) is a well-known treatable cause of refractory EPI [35] and thiamine deficiency is associated with EPI [49,50] and mouse model of EPI responds to thiamine supplementation [51].

The metabolomics findings of the DD are consistent with past studies. Specifically, in those with DD, we found involvement of ATP and other energy metabolites. Previous studies show elevations in the TCA and energy pathway metabolites in both urine [21] and plasma [13,14]. Studies examining the characteristics of those with ASD and mitochondrial disorder demonstrate a high incidence of DD, particularly motor delay [25]. Thus, those with ASD and DD may be particularly at risk for mitochondrial dysfunction. It is important to appreciate that the mitochondrial metabolites are in the center of many of the other physiological abnormalities identified. For example, mitochondria are integral to redox metabolism, calcium buffering, lipid homeostasis and steroid synthesis [25,52,53,54,55]. Furthermore, these mitochondrial metabolites are essential for amino acid regulation in the urea cycle and nitrogen disposal system. The nitrogen disposal system is partially located in the mitochondrial matrix where several amino acids, including alanine, lysine and the branched-chain amino acids (leucine, isoleucine and valine) are key metabolic intermediates of mitochondrial metabolism. Thus, dysfunction of the mitochondria can have a wide-reaching effect that can affect multiple systems and results in a general global DD rather than a disorder that affect specific systems. 

Although both energy and amino acid metabolism were found to be prominent abnormalities, the result of this analysis demonstrates the close interconnection between these pathways. For both EPI and DD, disruptions in amino acid pathways were disrupted primary by energy metabolism while for ASD the primary disruption to energy pathways was from disruption in amino acid pathway. Although amino acids are best known for their role in being the building blocks of proteins, they have critical roles in metabolism. As mentioned above, a prominent amino acid identified in our analysis in ASD is L-cysteine, particularly in its role in transsulfuration and redox metabolism in the production of GSH. The production of GSH, however, has other significant consequences since both glutamate and glycine are utilized along with cysteine to produce GSH. Both glycine and glutamate are also amino acid neurotransmitters with glutamate being the major excitatory neurotransmitter of the cortex. One of the common themes between ASD and EPI is the relative excitatory-inhibitory imbalance with a relative overabundance of glutamate [56]. A subgroup of patients with ASD have been identified in at least one study with abnormalities in relatively reduced concentrations of branched-chain amino acids [34] similar to branched-chain ketoacid dehydrogenase kinase deficiency which clinically presents with ASD, epilepsy, and intellectual disability [57]. Branched-chain amino acids have diverse physiological roles including modulating glucose and fatty acid metabolism as well as regulating important molecular pathways and promoting protein synthesis [58].

Our results have direct implications for treatment of ASD, DD and EPI. The dysregulation of L-cysteine in ASD points to interventions targeting the transmethylation and transsulfuration pathways, which have been shown to be responsive to such treatments as cobalamin [59] and N-acetylcysteine [60]. The DD group was found to have a deficit center in ATP production, which implicates the mitochondria. Treatments for mitochondria dysfunction are understudied but evidence favors standard treatment to support the mitochondria to improving energy production [26,61,62]. Abnormalities in NADP metabolism associated with EPI points to targeting the nicotinamide adenine dinucleotide precursors, which has been shown to have therapeutic biological effects on dyslipidemia [61], aging [62], Parkinson’s disease [63] and mitochondrial function [64].

The microbiome is being increasingly recognized for its role in neurodevelopmental disorders, particularly ASD [65]. Previous studies examining stool metabolites suggest that specific bacterial metabolites are associated with ASD [16]. Animal models of ASD demonstrate that propionate, an important short-chain fatty acid metabolite produced by gut bacteria, is associated with ASD-like behavior [66,67]. Studies have also demonstrated that both propionate [68] and butyrate [69] can modulate mitochondrial function in ASD cell lines. One of the questions that remains in this investigation of the gut–brain connection is whether metabolites from enteric bacteria can indeed reach the brain. 

In this study, we found two metabolites in CSF which are not produced in animal metabolism but rather by the metabolism of other microorganisms such as bacteria: shikimic acid was found to be associated with ASD, while *cis-cis*-muconic acid was associated with EPI. Shikimic acid is found in non-animal organisms including bacteria, fungi, algae, parasites, and plants, where it is an intermediate in the biosynthesis of the aromatic amino acids phenylalanine, tyrosine, and tryptophan, which are considered essential in humans. Shikimic acid is also thought to have anti-viral, anti-bacterial, anti-inflammatory and anti-fungal properties, which are utilized in drugs and cosmetic products [70]. Rodent studies have found that shikimic acid is protective in experimentally induced focal cerebral ischemia [71] and promotes oligodendrocyte precursor differentiation and accelerates remyelination [72]. The relative abundance of shikimic acid in our sample was 46.84 (standard deviation 63.5; 95% confidence interval 33.6–60.1). To investigate whether this finding could have been due to medication additive, we examined the 28 (31%) patients who demonstrated values outside of the upper limit of the confidence interval (i.e., >60.1). None of these patients were taking medications that included shikimic acid. 

*Trans-trans* muconic acid is found in human metabolism, but *cis-cis* muconic acid is not. *Cis,cis*-muconic acid is found in low quantities in most autotrophic organisms, allowing us to infer whether it was derived from the diet or produced by enteric bacteria or fungi in our sample. This compound is produced by microorganisms including *E. coli*, *Corynebacterium glutamicum*, *Pseudomonas putida*, and *Saccharomyces cerevisiae* via enzymatic degradation of various aromatic chemical compounds The relative abundance of *cis,cis*-muconic acid in our sample was 5870 (standard deviation 6724; 95% confidence interval 4464–7274). To investigate whether this finding could have been due to a medication additive, we examined the 30 (33%) patients who demonstrated values outside of the upper limit of the confidence interval (i.e., >7272). None of these patients were taking medications that included *cis,cis*-muconic acid. [73]. Thus, these findings suggest that enteric metabolites can enter the CSF and directly affect neurodevelopmental outcomes as suggested by previous studies [65].

## 4. Materials and Methods

### 4.1. Participants 

CSF specimens were obtained under the Biologic Materials Availability Program (BMAP) at Phoenix Children’s Hospital (PCH). The BMAP is a PCH Institutional Review Board (IRB)-approved (IRB# 09–054) tissue procurement system which makes biologic specimens available for research. Individuals participating in the BMAP undergo an informed consent for the collection and use of their biological specimens and information for future research. Parents or legally authorized representatives signed an informed consent form and participants sign an assent form if over 6 years of age and mentally able to understand the protocol. Participants were recruited from PCH (Phoenix, AZ, USA) prior to sample collection. The protocol was discussed with the families prior to neurosurgery. Samples were collected during central nervous system surgery by the neurosurgeon. 

The medical records of all patients with CSF samples available (*n* = 544) were reviewed for diagnoses of DD, ASD, and EPI as well for other significant medical conditions and medications. Those without DD or ASD were categorized as controls (CNT). Participant characteristics are provided in Table 2 below and Supplementary Table S3. ASD patients were used as the reference group for matching to other groups. Thirty-four patients with an ASD diagnosis were identified. These samples were matched to patient with 20 DD and 34 CNT based on several characteristics, including age at sample collection, gender and source of sample collection. Sample collection location was selected to be as close anatomically.

A total of 29% of the ASD group demonstrated genetic abnormalities with Trisomy X, 3q29 duplication, 22q13 deletion, 9q21.31–q.31.3 deletion and PTEN, NF1 and L1CAM mutations in one participant each, 16p12.2 deletions in two patients and one patient with a large 9q deletion along with 6p11.2 and 22q13.13 duplications. A total of 20% of participants in the DD group demonstrated genetic abnormalities with Trisomy X, Trisomy 8 mosaicism, a duplication on 16p11.2 and duplications on chromosomes 16 and 19 in one patient each. A total of 11% of participants with EPI demonstrated genetic abnormalities including Trisomy X and an NF1 mutation. None of the CNT participants demonstrated genetic abnormalities.

### 4.2. Sample Collections and Storage 

Immediately after collection, specimens were transported on wet ice to the biorepository laboratory and processed within 30 min or stored at 20 °C for a maximum of 24 h if processing was delayed. Samples were centrifuged at −5 °C at 4000× *g* RPMS for 15 min and divided into 1.0 mL aliquots. Aliquots were snap frozen immediately after processing and stored in Wheaton Cryo-vials at −196 °C in liquid nitrogen tanks. Our collection methods were designed to minimize any degradation in metabolites from temperature or processing methods [74]. Vials were labeled with a unique identifier. Information regarding sample characteristics were entered into Freezerworks Unlimited. 

### 4.3. Metabolomic Analysis

#### 4.3.1. Sample Processing

Frozen CSF samples were first thawed overnight under 4 °C, and 50 μL of each sample was placed in a 2 mL Eppendorf vial. The initial step for protein precipitation and metabolite extraction was performed by adding 500 μL MeOH and 50 μL internal standard solution (containing 1810.5 μM ^13^C_3_-lactate and 142 μM ^13^C_5_-glutamic acid). The mixture was then vortexed for 10 s and stored at −20 °C for 30 min, followed by centrifugation at 14,000× *g* RPM for 10 min at 4 °C. The supernatants (450 μL) were collected into a new Eppendorf vial, and dried using a CentriVap Concentrator (Labconco, Fort Scott, KS, USA). The dried samples were reconstituted in 150 μL of 40% PBS/60% ACN.

#### 4.3.2. Reagents 

LC–MS-grade acetonitrile (ACN), methanol (MeOH), ammonium acetate, and acetic acid were purchased from Fisher Scientific (Pittsburgh, PA, USA). Ammonium hydroxide was bought from Sigma-Aldrich (Saint Louis, MO, USA). DI water was provided in house by a Water Purification System from EMD Millipore (Billerica, MA, USA). PBS was bought from GE Healthcare Life Sciences (Logan, UT, USA). The standard compounds corresponding to the measured metabolites were purchased from Sigma-Aldrich (Saint Louis, MO, USA) and Fisher Scientific (Pittsburgh, PA, USA).

#### 4.3.3. Metabolic Method

The targeted LC–MS/MS method used here was modeled after that developed and used in a growing number of studies [75,76,77,78,79,80]. Briefly, all LC–MS/MS experiments were performed on an Agilent 1290 UPLC-6490 QQQ-MS (Santa Clara, CA, USA) system. Each sample was injected twice, 10 µL for analysis using the negative ionization mode and 4 µL for analysis using the positive ionization mode. Both chromatographic separations were performed in the hydrophilic interaction chromatography (HILIC) mode on a Waters XBridge BEH Amide column (150 × 2.1 mm^2^, 2.5 µm particle size, Waters Corporation, Milford, MA, USA). The flow rate was 0.3 mL/min, the auto-sampler temperature was kept at 4 °C, and the column compartment was set at 40 °C. The mobile phase was composed of Solvents A (10 mM ammonium acetate, 10 mM ammonium hydroxide in 95% H_2_O/5% ACN) and B (10 mM ammonium acetate, 10 mM ammonium hydroxide in 95% ACN/5% H_2_O). After the initial 1 min isocratic elution of 90% B, the percentage of Solvent B decreased to 40% at t = 11 min. The composition of Solvent B maintained at 40% for 4 min (t = 15 min), and then the percentage of Solvent B gradually went back to 90%, to prepare for the next injection. 

The mass spectrometer is equipped with an electrospray ionization (ESI) source. Targeted data acquisition was performed in the multiple-reaction-monitoring (MRM) mode. The whole LC–MS system was controlled by Agilent Masshunter Workstation software (Santa Clara, CA, USA). The extracted MRM peaks were integrated using Agilent MassHunter Quantitative Data Analysis (Santa Clara, CA, USA). A pooled sample, which was a mixture of all CSF samples, was used as the quality control (QC) sample. We ran the QC once every 10 study samples to ensure good data quality.

### 4.4. Data Analysis

Metabolomics data were reviewed and compounds with >20% coefficient of variation without measurable peaks were eliminated. For remaining compounds, zero values were replaced with 0.1 to allow for log transformation of the data. MetaboAnalyst 5.0 was used for data analysis [81]. Data were normalized using log_10_ transformation (See Supplementary Figure S1).

Linear model analysis was used to define the significantly different metabolites from the three disease groups (ASD, DD, EPI) as compared to the CNT group by conducting three analyses. Each of the three analyses specified one of the disease groups as the comparison group and the other two disease groups as covariates. In this way, metabolites specific to each of the disease groups were found while controlling for the other disease groups. Alpha was set to ≤0.05. Heatmaps of significant metabolites were created using autoscale features standardization, Euclidean distance and the Ward clustering algorithm. 

Key metabolites identified by linear models for each clinical group were investigated using the metabolite–metabolite interaction tool of the network analysis package MetaboAnalyst 5.0. Log FC for each significant compound was entered into the analysis. Graphs demonstrating the z-value derived from the *p*-value and topology values were graphed for each clinical group with the prominent pathways (defined as *p* or topology z-value ≥ 2SD) for each neurodevelopmental disorder group labeled. A topology-based method was used since it takes advantage of dependencies and interactions between metabolites to enhance the biological relevance of the analysis [82]. Pathways were categorized into eight major categories based on their KEGG designation and biological mechanisms. The overlaps between pathways identified were analyzed and displayed in a Venn diagram and the percentage of pathways in each category was compared to the total number of pathways for each disorder in a bar graph. 

Metabolite networks were then analyzed in detail. First, the major key metabolites were determined by decreasing node scope to minimum number of nodes to the top-level nodes. To understand how the major metabolites interact with pathways identified as well as to understand the interaction of metabolites within the pathways, pathways within the defined category were selected to view.

## 5. Conclusions

This study examined the metabolomics of CSF in three neurodevelopmental conditions, ASD, DD and EPI, using linear regression and metabolite–metabolite pathway analysis. Energy and amino acid pathways were commonly disrupted in all conditions, with redox pathways being relatively more disrupted in ASD and vitamin and one-carbon pathways being disrupted in DD and EPI and lipid pathways being disrupted in EPI. Despite these shared pathways, the major core nodes driving the metabolic disruption were different for each disorder: L-cysteine, adenine and dodecanoic disruption for ASD; NADP for EPI and ATP for DD. Interestingly, two microbiome metabolites were found with significant concentrations in the CSF: shikimic acid and *cis-cis*-muconic acid. 

This study had several limitations including selection of samples from a general biorepository which was not specifically designed to characterize these neurodevelopmental disorders. Another limitation includes the fact that the age of diagnosis of ASD, DD and EPI can vary. Although these disorders are believed, in general, to be due to processes that occurred prenatally, the age of onset of symptoms can vary. Thus, the findings of this study should guide future studies which prospectively collect CSF samples along with dense clinical information and larger sample sizes to better characterize the patients and match on age of onset of symptomatology. In addition, simultaneous collection of blood, urine and stool samples may be very useful to better understand how systematic and microbiome changes in metabolism can be transmitted to the CSF and potentially affect brain function.

## Figures and Tables

**Table 1:** Previous metabolomics studies on neurologic and neurodevelopmental disorders.

	Study	Groups	*n*	Ages	Findings
**Blood**					
**Blood**	Murgia 2017 [10]	EPI “responder” EPI “non-responder” CNT	18 17 35	47.5 y 51.17 y 44.68 y	Elevated concentrations of 2-OH-valerate, 2-OH-butyrate, acetoacetate, acetone, acetate, choline, alanine, glutamate, scyllo-inositol and decreased concentrations of glucose, lactate, citrate were found in EPI patients.
	Wei 2012 [11]	EPI	19	10–40 y	Increased L-glutamate, glycine, glyceric acid, lactic acid, inositol, myristic acid and decreased GABA, creatine, L-thronine, L-tryptophan in patients with EPI.
		CNT	33	20–29 y	
	Wang 2016 [12]	EPI	27	~35.1 y	EPI patients with seizures had elevated lactate, butanoic acid, proline, L-glutamate and lower palmitic acid, linoleic acid, elaidic acid, trans-13-octadecenoic acid, stearic acid, citrate cysteine, glutamine, asparagine, glyceraldehyde.
		CNT	23	~37.6 y	
	**Plasma**				
**Plasma**	Orozco 2019 [13]	ASD	167	24–60 m	Elevated alanine, glycine, ornithine, serine in ASD. Elevated acetate, glutamate, lactate, and TCA cycle intermediates in D.
		DD	51		
		DS	31		
		CNT	193		
	Sotelo-Orozco 2020 [14]	ASD	167	24–60 m	Metabolites in energy metabolism including lactate, pyruvate, ketone bodies (3-hydroxybutyrate and acetoacetate), Kreb cycle metabolites (cis-aconitate and fumarate), and ornithine were associated with cognitive function, adaptive skills, and aberrant behaviors.
		DD	51		
		DS	31		
		TD	193		
	Needham 2021 [15]	ASD	57	3–12 y	Elevated short-chain acylcarnitines and lower long-chain acylcarnitines in ASD.
		TD	40		
	**Stool**				
**Stool**	Needham 2021 [15]	ASD	57	3–12 y	Elevated acetylcarnitines (C2) and carnitine in ASD.
		TD	40		
	Kang 2018 [16]	ASD	21	4–17 y	Isopropanol elevated in ASD.
		CNT	23		
	De Angelis 2013 [17]	ASD	10	4–10 y	Alterations in phenol compounds in ASD.
		DD	10		
		CNT	10		
	Qureshi 2020 [18]	ASD	18	16–17 y	Alterations in indole in ASD.
		TD	20		
	**Urine**				
**Urine**	Ming 2012 [19]	ASD	48	~10 y	Lower amino acid concentrations in ASD. Propionate was related to gastrointestinal symptoms in children with ASD.
		CNT	53		
	Gevi 2020 [20]	ASD	40	4–5 y	Abnormalities in monoamine neurotransmitters, 4-cresol and pyridoxal-5-phosphate, in children with ASD.
		CNT	40		
	Chen 2021 [21]	GDD	863	N/A	Found elevated concentrations glycolyic, 3-hydroxyisobutyric acid and lower levels of palmitic acid common to both GDD and ID group.
		ID	367		
		CNT	100		
	**Cerebrospinal Fluid (CSF)**				
**CSF**	Akiyama 2020 [22]	EPI	34	0–17 y	EPI patients had elevated concentrations of pyridoxamine, tyrosine and reduced concentrations of 2-ketoglutaric acid, 1,5-anhydroglucitol.
		CT	30		
	**Post-Mortem Brain**				
**Post-Mortem Brain**	Graham 2020 [23]	ASD	11	Age matched	ASD individuals showed disruptions in pyrimidine, ubiquinone and vitamin K metabolism and elevations in long-chain fatty acids.
		CT	10		
	Lalwani 2020 [24]	EPI	15	~40.67 y	Alterations in fatty acid and pentose phosphate pathways, vitamin metabolism including thiamine, one-carbon, nicotinamide, pantothenate and CoA pathways and amino acid metabolism including phenylalanine and tyrosine in EPI.
		CT	15	~40.8 y	

ASD = autism spectrum disorder, DD = developmental delay, EPI = epilepsy, CNT = control, DS = Down syndrome, GDD = global developmental delay, ID = intellectual disability, m = month, TD = typically developing, y = year.

**Table 2:** Patient demographics and co-occurring conditions.

	ASD	Developmental Delay	Control	Epilepsy *
**Demographics**				
N	34	20	34	18
Age Mean (St Dev)	7.3 y (4.6 y)	6.4 y (4.7 y)	7.2 y (4.5 y)	7.0 y (4.8 y)
Sex (%Male)	82%	60%	79%	72%
**Co-Occurring Conditions**				
Hydrocephalus	26%	15%	26%	17%
Macrocephaly	6%	0%	0%	6%
Microcephaly	3%	5%	0%	0%
CNS Cysts/Tumors	24%	45%	9%	33%
CNS Malformation	26%	10%	41%	22%
Tethered Cord	29%	30%	44%	17%
Chiari Malformation	21%	60%	3%	50%
Intraventricular Hemorrhage	6%	0%	3%	0%
Traumatic Brain Injury	9%	0%	15%	0%
Chronic Headache/Migraine	0%	30%	6%	11%
Cerebral Palsy	9%	15%	0%	11%
Premature	6%	10%	15%	6%
Learning and Behavior Disorders	64%	70%	0%	44%
Intellectual Disability	26%	5%	0%	22%
Genetic Abnormality	29%	20%	0%	11%
In Utero Exposure	6%	10%	6%	6%
Asthma/Respiratory Issues	12%	15%	9%	11%
Movement/Coordination Disorders	21%	20%	6%	6%
Sleep Disorder	18%	15%	21%	17%
Gastrointestinal Disorders	59%	20%	15%	17%
Kidney/Urological Issues	6%	5%	0%	6%
Immune Disorders	6%	10%	6%	11%
Congenital Heart Condition	9%	10%	6%	6%
Non-Congenital Heart Condition	6%	5%	3%	0%

y = years. * patients with epilepsy are also part of the other three groups.

## Data Availability

Data is contained within the article.

## Supplementary Materials

The following supporting information can be downloaded at: https://www.mdpi.com/article/10.3390/metabo12050371/s1, Figures S1–S7: Data Normalization for Individual Features and Metabolite–Metabolite Interaction Networks for Specific Pathways; Table S1: Significant Metabolites for each Neurodevelopmental Group; Table S2: Metabolite–Metabolite Pathway Analysis; Table S3: Participant Characteristics and Group Matching.
