# Supplementary material for: Central Nervous System Metabolism in Autism, Epilepsy and Developmental Delays: A Cerebrospinal Fluid Analysis
Source: Metabolites. 2022 Apr 20;12(5):371. doi: 10.3390/metabo12050371 (PMC9148155; doi:10.3390/metabo12050371)
Supplement: Supplementary file 1 [file metabolites-12-00371-s001.zip › Supplementary Figures.pdf]

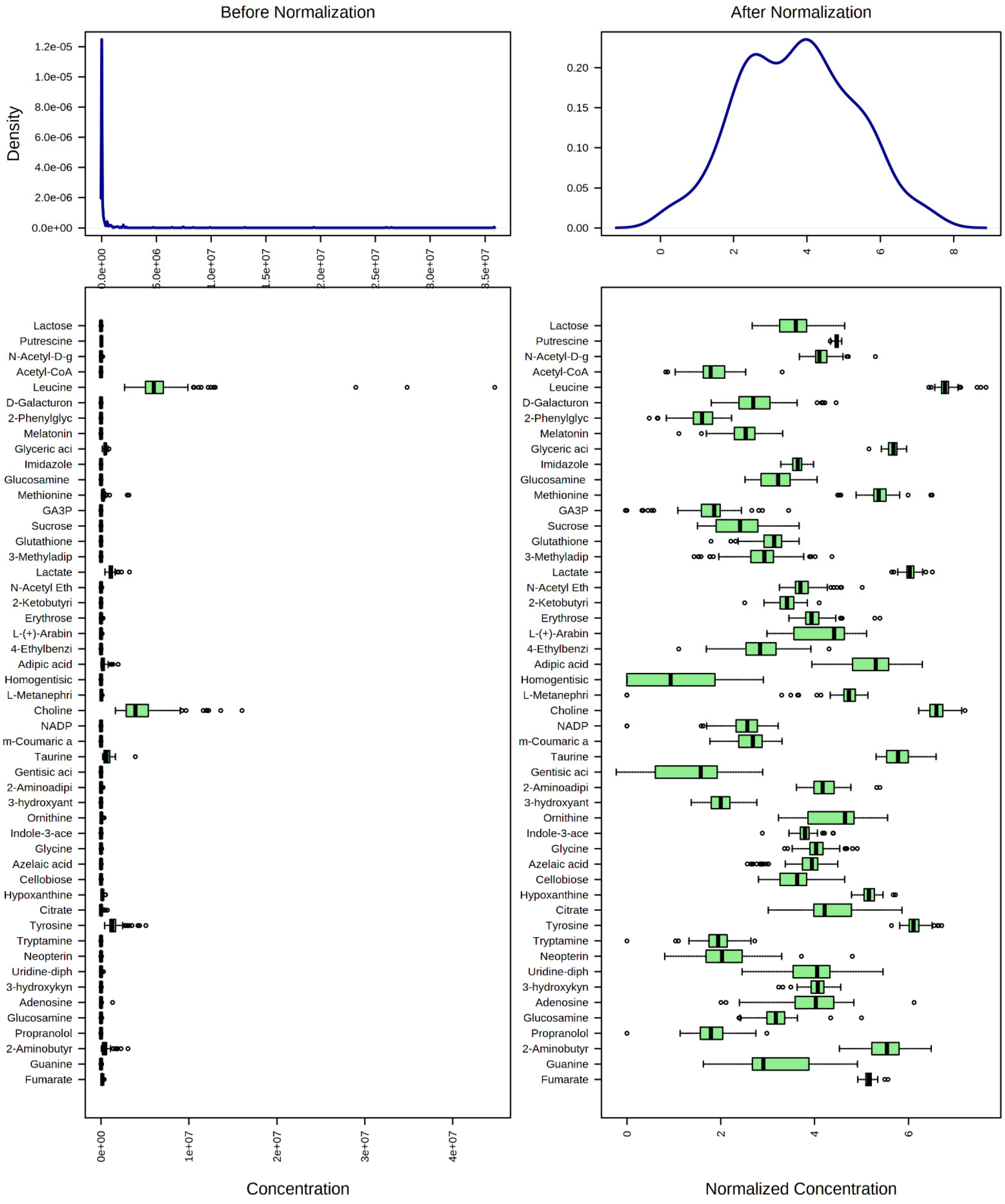

Figure S1. Data Normalization for Individual Features.

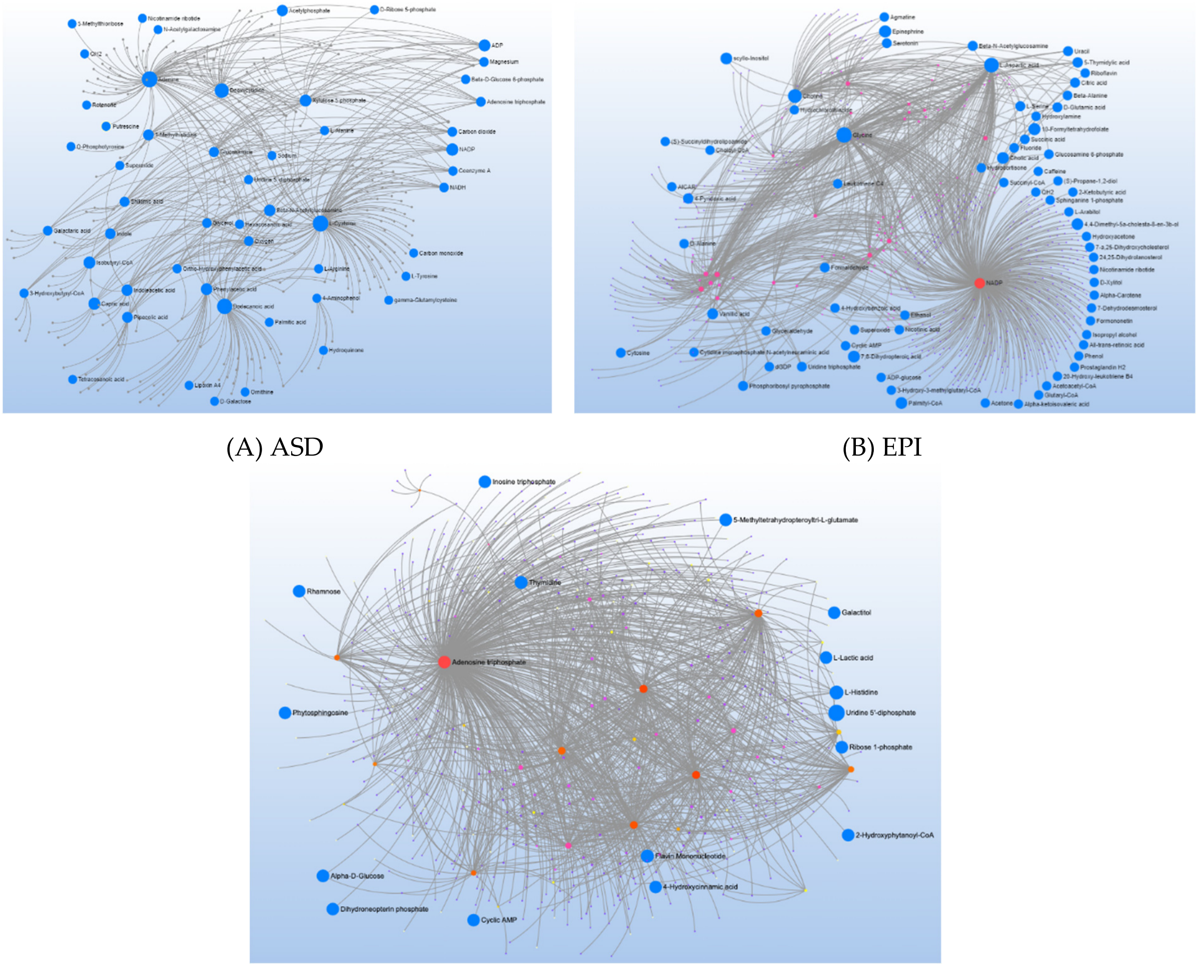

(A) ASD

(B) EPI

(C) DD

Figure S2. Neurotransmitter Pathways for Metabolite-Metabolite Interaction Networks for (a) ASD, (b) Epilepsy and (c) DD.

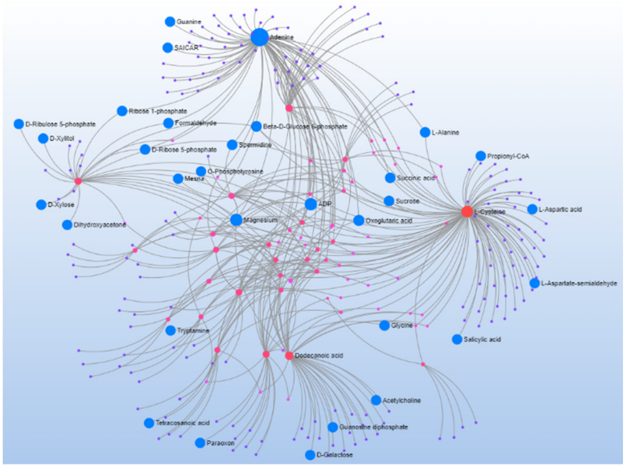

(A) ASD

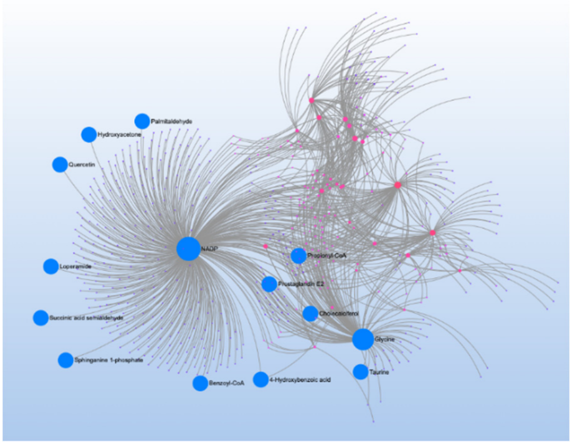

(B) EPI

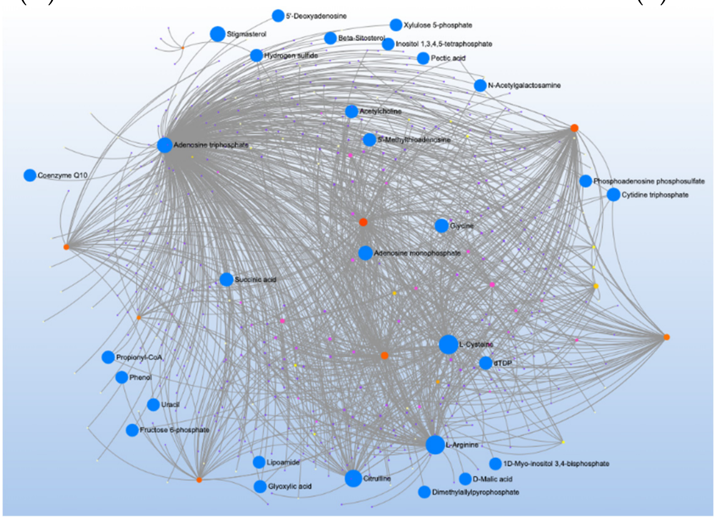

(C) DD

Figure S3. Redox Pathways for Metabolite-Metabolite Interaction Networks for (a) ASD, (b) Epilepsy and (c) DD.

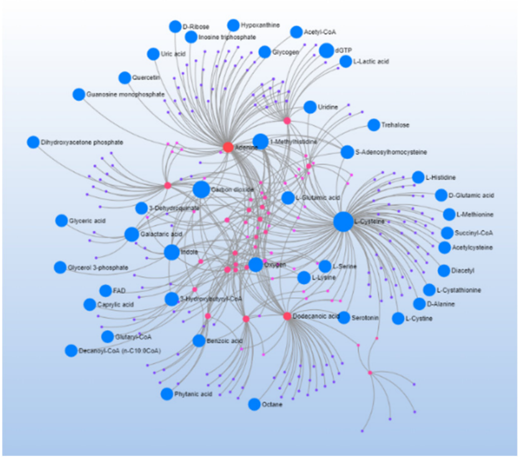

(A) ASD

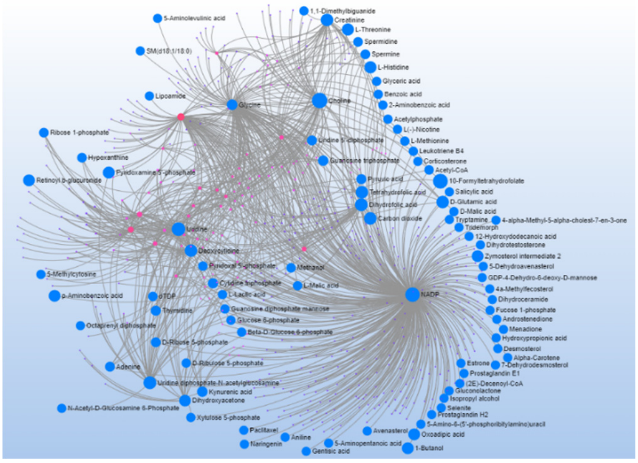

(B) EPI

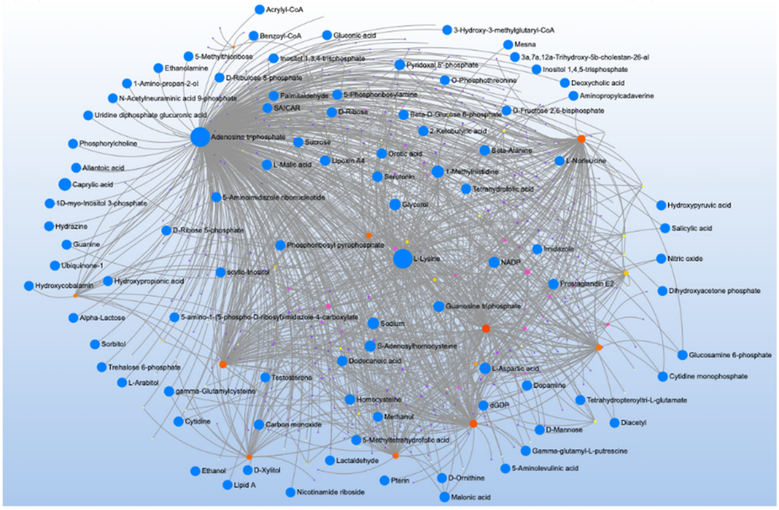

(C) DD

Figure S4. Nucleotide Pathways for Metabolite-Metabolite Interaction Networks for (a) ASD, (b) Epilepsy and (c) DD.

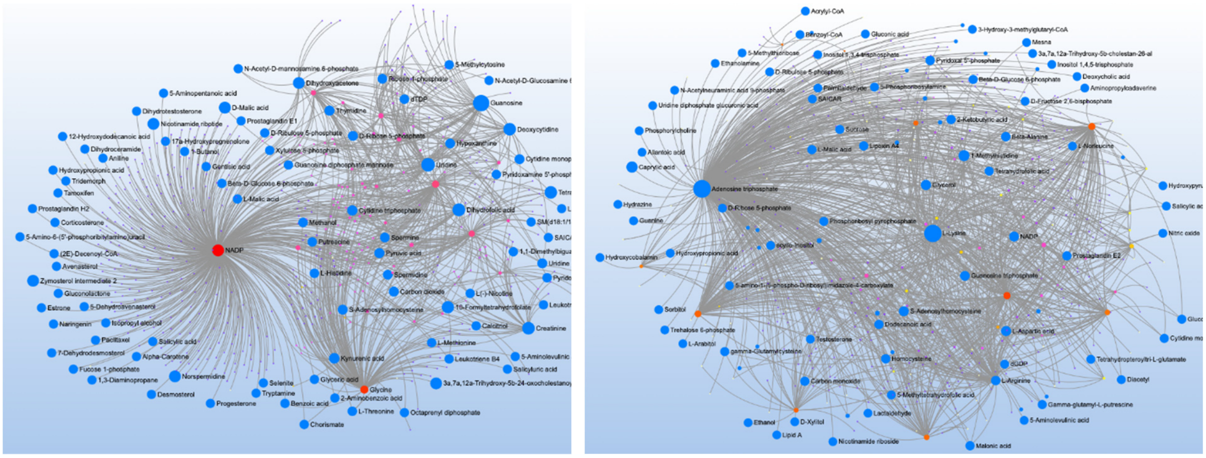

(A) EPI (B) DD

Figure S5. One-Carbon Metabolism Pathways for Metabolite-Metabolite Interaction Networks for (a) Epilepsy and (b) DD.

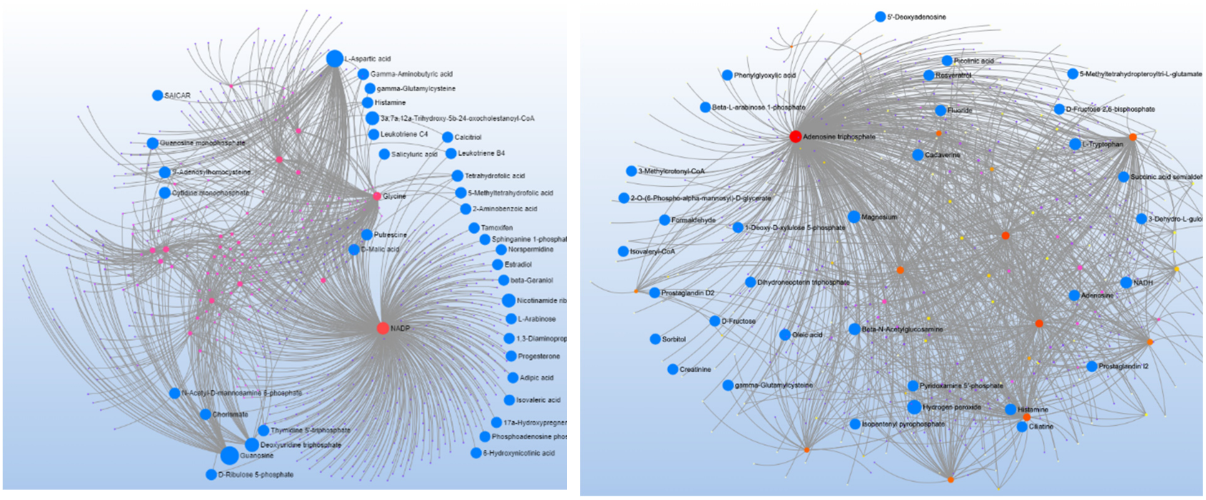

(A) EPI (B) DD

Figure S6. Vitamin Pathways for Metabolite-Metabolite Interaction Networks for (a) Epilepsy and (b) DD.

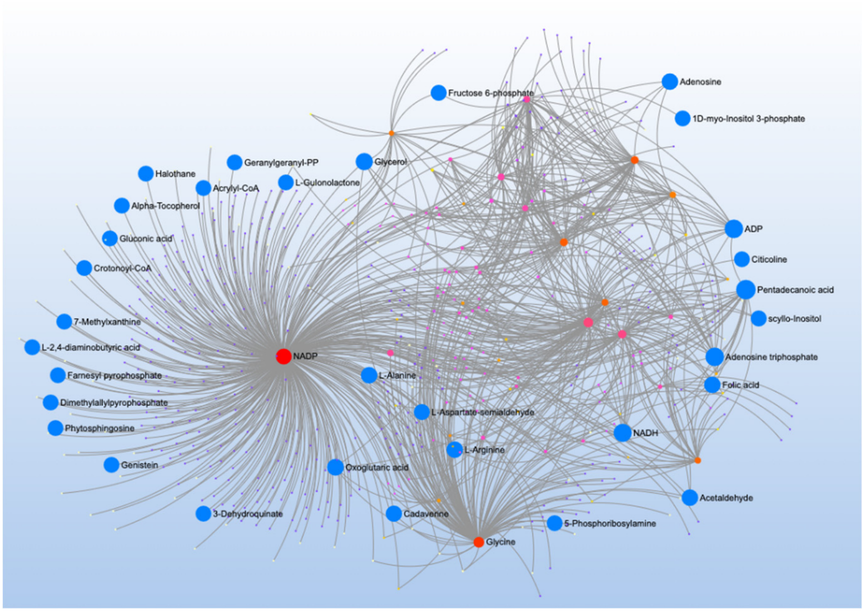

(A) EPI

Figure S7. Lipid Pathways for Metabolite-Metabolite Interaction Networks for (b) Epilepsy.
