# Supplementary material for: Central Nervous System Metabolism in Autism, Epilepsy and Developmental Delays: A Cerebrospinal Fluid Analysis
Source: Metabolites. 2022 Apr 20;12(5):371. doi: 10.3390/metabo12050371 (PMC9148155; doi:10.3390/metabo12050371)
Supplement: Supplementary file 1 [file metabolites-12-00371-s001.zip › Supplementary Table S1.pdf]

ASD				Epilepsy				Developmental Delay			
Metabolite	t value	p value	Fold Change	Metabolite	t value	p value	Fold Change	Metabolite	t value	p value	Fold Change
Isobutyryl-CoA	6.86	1.01E-09	2.1	Cytidine	3.79	0.000282	1.5	Isobutyryl-CoA	-3.35	0.001214	0.7
3-Hydroxybutyryl-CoA	4.11	8.89E-05	1.2	Acetylglucosamine	3.53	0.00066	1.2	Cystamine	3.16	0.002151	1.8
Lauric acid	3.49	0.000766	1.2	Deoxycytidine	3.14	0.002326	1.5	Methylmalonic acid	2.96	0.004026	1.3
Shikimic acid	3.39	0.001062	2.2	cis-cis-Muconic acid	2.95	0.004129	1.7	ATP	2.80	0.006268	1.4
Cysteine	3.13	0.002388	1.7	Guanosine	2.92	0.00442	1.5	4-Aminophenol	2.74	0.007554	1.3
4-Aminophenol	-2.96	0.003976	0.7	N-Acetyl-D-galactosamine	2.81	0.006052	1.2	Cysteine	-2.66	0.009229	0.6
Deoxycytidine	-2.87	0.005222	0.7	Choline	2.79	0.006572	1.2	1-Methylhistidine	-2.52	0.013465	0.8
Benzoic acid	2.83	0.005748	1.3	5-Methylcytidine	2.77	0.006872	1.3	DCDP	2.43	0.017032	1.5
Glucosamine	2.69	0.008533	1.3	Xanthosine	2.72	0.007804	1.2	Stachyose	-2.39	0.018979	0.7
N,N-Dicyclohexylurea	2.65	0.009554	1.1	Phenylbutazone	2.62	0.010451	1.4	Cytosine	2.26	0.026544	1.6
Cystamine	-2.62	0.010376	0.6	Aspartate	2.59	0.011316	1.3	N-Acetyl-D-galactosamine	2.25	0.026811	1.1
Adenine	2.46	0.016075	1.5	Uridine-diphosphate-N-acetylglucosamine	2.40	0.018345	1.5	Homoserine	2.18	0.032132	1.1
Mucic acid	2.44	0.01668	1.4	Glycine	2.38	0.019475	1.2	Arginine	2.18	0.032267	1.1
GA3P	-2.43	0.017232	0.7	Gibberellic acid	2.33	0.022273	1.7	Citrulline	2.16	0.033891	1.2
1-Methylhistidine	2.40	0.018494	1.3	Kynurenic acid	2.32	0.022824	1.6	Ornithine	2.12	0.036705	1.4
malonic acid	2.36	0.020304	1.2	Dihydrofolic acid	-2.26	0.026578	0.8	Lysine	2.11	0.037461	1.1
Nonadecanoic acid	2.31	0.023427	1.1	Pentadecanoic acid	2.21	0.029486	1.1	Pyroglutamic acid	2.06	0.042392	1.1
Indole	2.29	0.024207	1.1	Dihydroxyacetone	2.19	0.031141	1.1	Glutamine	2.06	0.042703	1.1
Capric acid	2.29	0.024431	1.2	D-Xylonic acid	2.17	0.032412	1.2	Asparagine	2.04	0.044111	1.1
Methylguanidine	-2.23	0.028097	0.8	N-Acetylglutamine	2.17	0.032699	1.2				
Phosphocreatine	2.15	0.034643	1.1	Creatinine	2.11	0.037395	1.6				
Heptadecanoic acid	2.09	0.039531	1.3	L-Normetanephrine	2.08	0.040446	1.1				
2-Hydroxyphenylacetic acid	2.08	0.040262	1.3	NADP	-2.06	0.042911	0.8				
Fumarate	-2.07	0.041132	0.9	Uridine	2.04	0.044366	1.2				
Phenylacetic acid	-2.06	0.042606	0.9	dUTP	-2.04	0.044519	0.8				
D-xylulose 5-phosphate	2.06	0.042669	1.7	Galactonic acid	2.01	0.047895	1.2				
Indole-3-acetic acid	2.04	0.044147	1.1	Vanillic acid	2.00	0.048703	1.4				
Pipecolinic acid	2.01	0.048089	1.2	4-Pyridoxic acid	2.00	0.049052	1.1				
