# Supplementary material for: Central Nervous System Metabolism in Autism, Epilepsy and Developmental Delays: A Cerebrospinal Fluid Analysis
Source: Metabolites. 2022 Apr 20;12(5):371. doi: 10.3390/metabo12050371 (PMC9148155; doi:10.3390/metabo12050371)
Supplement: Supplementary file 1 [file metabolites-12-00371-s001.zip › Supplementary Table S2.pdf]

Supplementary Table S2. Metabolite-Metabolite Pathway Analysis for ASD, Epilepsy and Developmental Delay Clinical Groups

ASD Pathway Analysis

Pathway	Total	Expected	Hits	P.Value	Topology	PVal.Z	Topo.Z	General Pathway Category
Purine metabolism	65	7.75	27	5.08E-10	1.55	3.9	1.91	Nucleotide
Aminoacyl-tRNA biosynthesis	48	5.73	21	1.70E-08	0.489	3.15	-0.043	Amino Acid
Pyrimidine metabolism	39	4.65	18	7.06E-08	1.79	2.84	2.36	Nucleotide
Glutathione metabolism	28	3.34	14	6.53E-07	1.7	2.37	2.2	Redox Metabolism
Glyoxylate and dicarboxylate metabolism	32	3.82	15	7.37E-07	0.742	2.34	0.423	Energy Metabolism
Cysteine and methionine metabolism	33	3.94	15	1.21E-06	1.16	2.23	1.19	Redox Metabolism
Pantothenate and CoA biosynthesis	19	2.27	10	1.63E-05	1.06	1.68	1	Energy Metabolism
Phenylalanine metabolism	10	1.19	7	2.71E-05	1.67	1.57	2.13	Amino Acid, Neurotransmitter
Valine, leucine and isoleucine biosynthesis	8	0.954	6	6.09E-05	0.857	1.4	0.636	Amino Acid, Energy Metabolism
Glycine, serine and threonine metabolism	33	3.94	12	0.000215	1.12	1.13	1.13	Amino Acid, Neurotransmitter
Glycolysis or Gluconeogenesis	26	3.1	10	0.000438	1	0.974	0.9	Energy Metabolism
Pentose phosphate pathway	22	2.62	9	0.000502	1.24	0.945	1.34	Energy Metabolism
Arginine biosynthesis	14	1.67	7	0.000508	1.38	0.942	1.61	Amino Acid
Butanoate metabolism	15	1.79	7	0.000857	0.929	0.83	0.768	Energy Metabolism
Alanine, aspartate and glutamate metabolism	28	3.34	10	0.000872	1.07	0.826	1.04	Amino Acid, Energy Metabolism Redox, Neurotransmitter
beta-Alanine metabolism	21	2.5	8	0.00181	0.95	0.67	0.808	Energy Metabolism
Tryptophan metabolism	41	4.89	12	0.002	0.75	0.649	0.438	Neurotransmitter, Amino Acid
D-Glutamine and D-glutamate metabolism	6	0.716	4	0.00242	1.2	0.608	1.27	Amino Acid
Propanoate metabolism	23	2.74	8	0.00354	0.818	0.527	0.564	Energy Metabolism
Phenylalanine, tyrosine and tryptophan biosynthesis	4	0.477	3	0.0061	2.33	0.41	3.36	Neurotransmitter, Amino Acid
Citrate cycle (TCA cycle)	20	2.39	7	0.00611	0.895	0.41	0.706	Energy Metabolism
Taurine and hypotaurine metabolism	8	0.954	4	0.00931	1	0.32	0.9	Redox Metabolism, Energy Metabolism
Valine, leucine and isoleucine degradation	40	4.77	10	0.0155	0.513	0.211	0.000332	Amino Acid, Energy Metabolism
Nitrogen metabolism	6	0.716	3	0.0254	0.6	0.105	0.161	Amino Acid
Arginine and proline metabolism	38	4.53	9	0.03	0.784	0.0701	0.501	Amino Acid
Histidine metabolism	16	1.91	5	0.0332	0.667	0.0479	0.284	Amino Acid
Glycerolipid metabolism	16	1.91	5	0.0332	0.6	0.0479	0.161	Energy Metabolism
Pyruvate metabolism	22	2.62	6	0.0385	0.714	0.0165	0.372	Energy Metabolism

Epileptic Pathway Analysis

Pathway	Total	Expected	Hits	P.Value	Topology	PVal.Z	Topo.Z	General Pathway Category
Pyrimidine metabolism	39	9.5	32	2.30E-14	2.82	4.66	2.36	Nucleotide, One-Carbon
Purine metabolism	65	15.8	42	2.27E-12	2.12	3.89	1.52	Nucleotide, One-Carbon
Glycine, serine and threonine metabolism	33	8.04	25	4.70E-10	1.84	2.99	1.18	Amino Acid, Neurotransmitter
Glyoxylate and dicarboxylate metabolism	32	7.8	22	1.05E-07	1.29	2.08	0.511	Energy Metabolism
Alanine, aspartate and glutamate metabolism	28	6.82	20	1.54E-07	1.81	2.02	1.15	Amino Acid, Energy Metabolism Redox, Neurotransmitter
beta-Alanine metabolism	21	5.12	16	7.05E-07	1.6	1.76	0.887	Energy Metabolism
Arginine biosynthesis	14	3.41	12	2.13E-06	2.08	1.58	1.47	Amino Acid
One carbon pool by folate	9	2.19	9	2.82E-06	4.5	1.53	4.41	Vitamin, One-Carbon
Steroid biosynthesis	42	10.2	23	1.75E-05	1.07	1.22	0.247	Lipid Metabolism
Pentose phosphate pathway	22	5.36	14	9.45E-05	1.57	0.939	0.852	Energy Metabolism
Propanoate metabolism	23	5.61	14	0.000189	1.36	0.823	0.6	Energy Metabolism
Phenylalanine metabolism	10	2.44	8	0.000328	1.44	0.73	0.698	Amino Acid, Neurotransmitter
Butanoate metabolism	15	3.66	10	0.000599	1.5	0.629	0.765	Energy Metabolism
Aminoacyl-tRNA biosynthesis	48	11.7	22	0.000796	0.532	0.581	-0.409	Amino Acid
Pantothenate and CoA biosynthesis	19	4.63	11	0.00172	1	0.452	0.159	Vitamin, Energy Metabolism
Glutathione metabolism	28	6.82	14	0.00269	1.59	0.376	0.878	Redox
Citrate cycle (TCA cycle)	20	4.87	11	0.00299	1.42	0.358	0.67	Energy Metabolism
Valine, leucine and isoleucine biosynthesis	8	1.95	6	0.00359	0.857	0.328	-0.0147	Amino Acid, Energy Metabolism
Pentose and glucuronate interconversions	18	4.39	10	0.00423	0.941	0.3	0.0873	Energy Metabolism
Tryptophan metabolism	41	9.99	18	0.00429	1.05	0.298	0.219	Amino Acid, Neurotransmitter
Arginine and proline metabolism	38	9.26	17	0.00432	1.16	0.297	0.355	Amino Acid
Vitamin B6 metabolism	9	2.19	6	0.00857	2.38	0.182	1.83	Vitamin
Glycolysis or Gluconeogenesis	26	6.34	12	0.012	1.08	0.126	0.256	Energy Metabolism
Folate biosynthesis	27	6.58	12	0.0169	1.31	0.0676	0.532	Vitamin, One-Carbon
Pyruvate metabolism	22	5.36	10	0.024	1.14	0.00832	0.332	Energy Metabolism
D-Glutamine and D-glutamate metabolism	6	1.46	4	0.0341	1.2	-0.0504	0.401	Amino Acid
Nitrogen metabolism	6	1.46	4	0.0341	0.8	-0.0504	-0.0841	Amino Acid
Amino sugar and nucleotide sugar metabolism	37	9.02	14	0.0456	0.861	-0.0993	-0.00992	Nucleotide
Riboflavin metabolism	4	0.975	3	0.0471	3	-0.105	2.59	Vitamin
Phenylalanine, tyrosine and tryptophan biosynthesis	4	0.975	3	0.0471	2	-0.105	1.37	Amino Acid, Neurotransmitter
Sphingolipid metabolism	21	5.12	9	0.0473	1.2	-0.106	0.401	Lipid Metabolism

Developmental Delay Pathway Analysis

Pathway	Total	Expected	Hits	P.Value	Topology	PVal.Z	Topo.Z	General Pathway Category
Purine metabolism	65	14.3	44	8.60E-16	2.12	4.7	1.51	Nucleotide, One-Carbon
Pyrimidine metabolism	39	8.6	31	1.54E-14	2.82	4.25	2.34	Nucleotide, One-Carbon
Glycine, serine and threonine metabolism	33	7.28	24	4.73E-10	1.78	2.67	1.09	Amino Acid, Neurotransmitter
Glyoxylate and dicarboxylate metabolism	32	7.06	23	1.64E-09	1.29	2.47	0.501	Energy Metabolism
Alanine, aspartate and glutamate metabolism	28	6.17	19	2.08E-07	1.63	1.73	0.91	Amino Acid, Energy Metabolism
Arginine biosynthesis	14	3.09	12	6.64E-07	2.08	1.55	1.45	Amino Acid
beta-Alanine metabolism	21	4.63	15	1.59E-06	1.6	1.42	0.874	Energy Metabolism
Arginine and proline metabolism	38	8.38	20	2.85E-05	1.32	0.974	0.542	Amino Acid
One carbon pool by folate	9	1.98	8	3.81E-05	4.25	0.929	4.07	One-Carbon, Vitamin
Glutathione metabolism	28	6.17	16	4.98E-05	1.78	0.888	1.09	Redox
Propanoate metabolism	23	5.07	14	5.79E-05	1.36	0.865	0.59	Energy Metabolism
Pantothenate and CoA biosynthesis	19	4.19	12	0.000123	1.28	0.749	0.486	Energy Metabolism, Vitamin
Pentose phosphate pathway	22	4.85	13	0.000166	1.52	0.703	0.783	Energy Metabolism
Aminoacyl-tRNA biosynthesis	48	10.6	22	0.000171	0.532	0.698	-0.412	Amino Acid
Amino sugar and nucleotide sugar metabolism	37	8.16	18	0.000272	1	0.627	0.152	Nucleotide
Citrate cycle (TCA cycle)	20	4.41	11	0.00124	1.42	0.393	0.659	Energy Metabolism
Glycolysis or Gluconeogenesis	26	5.73	13	0.00145	1.2	0.37	0.393	Energy Metabolism
Phenylalanine metabolism	10	2.21	7	0.00154	1.67	0.36	0.955	Amino Acid, Neurotransmitter
Pentose and glucuronate interconversions	18	3.97	10	0.00189	1	0.329	0.152	Energy Metabolism
Valine, leucine and isoleucine biosynthesis	8	1.76	6	0.00206	0.857	0.316	-0.0205	Amino Acid, Energy Metabolism
Cysteine and methionine metabolism	33	7.28	15	0.00214	1.16	0.31	0.34	Amino Acid, Redox, One-Carbon
Galactose metabolism	27	5.95	13	0.00224	0.962	0.303	0.105	Energy Metabolism
Vitamin B6 metabolism	9	1.98	6	0.00504	2.62	0.178	2.11	Vitamin
Inositol phosphate metabolism	30	6.62	13	0.00696	0.931	0.129	0.0685	Energy Metabolism
Valine, leucine and isoleucine degradation	40	8.82	16	0.00727	0.949	0.122	0.0898	Amino Acid, Energy Metabolism
Butanoate metabolism	15	3.31	8	0.00765	1.21	0.114	0.41	Energy Metabolism
Pyruvate metabolism	22	4.85	10	0.0119	1.14	0.046	0.324	Energy Metabolism
Fructose and mannose metabolism	20	4.41	9	0.0182	0.895	-0.0192	0.0248	Energy Metabolism
D-Glutamine and D-glutamate metabolism	6	1.32	4	0.0239	1.2	-0.0609	0.393	Amino Acid
Nitrogen metabolism	6	1.32	4	0.0239	0.8	-0.0609	-0.0893	Amino Acid
Nicotinate and nicotinamide metabolism	15	3.31	7	0.0295	1.5	-0.0935	0.754	Vitamin
Riboflavin metabolism	4	0.882	3	0.0356	3	-0.122	2.56	Vitamin
Phenylalanine, tyrosine and tryptophan biosynthesis	4	0.882	3	0.0356	2.33	-0.122	1.76	Amino Acid, Neurotransmitter
Neomycin, kanamycin and gentamicin biosynthesis	2	0.441	2	0.0485	2	-0.17	1.36	
