# Supplementary material for: Central Nervous System Metabolism in Autism, Epilepsy and Developmental Delays: A Cerebrospinal Fluid Analysis
Source: Metabolites. 2022 Apr 20;12(5):371. doi: 10.3390/metabo12050371 (PMC9148155; doi:10.3390/metabo12050371)
Supplement: Supplementary file 1 [file metabolites-12-00371-s001.zip › Supplementary Table S3.pdf]

Table S3. Patient characteristics for three neurodevelopmental groups, including age at sample acquisition, sex, source of sample collection, history of epilepsy and perioperative medications.

**Autism Spectrum Disorder**					**Developmental Delay**					**Control**				
**Age**	**Sex**	**Source**	**Epi**	**Perioperative Medications**	**Age**	**Sex**	**Source**	**Epi**	**Perioperative Medications**	**Age**	**Sex**	**Source**	**Epi**	**Perioperative Medications**
15y	F	PF	N	levothyroxine, metformin, gabapentin, amitriptyline, hydrocodone, cefazolin, litramine, balsalazide, diazepam, clonidine, lidocaine, oxycodone, propofol, fentanyl, dexmedetomidine, dexamethasone, ondansetron, ketamine, midazolam, cefazolin	14y	F	BV	N	lisinopril, ibuprofen, dexmedetomidine, pantoprazole, ketorolac, acetazolamide, docusate, fentanyl, rocuronium	15y	F	PF	N	fentanyl, propofol, rocuronium, lidocaine, metoclopramide, Ondansetron, midazolam, cyproheptadine, naproxen, alprazolam
4y	M	BV	Y	ranitidine, lacosamide, clonazepam, fentanyl, dexmedetomidine, dexamethasone, rocuronium	4y	M	PF	N	Cefazolin, rocuronium, fentanyl, propofol, ondansetron, dexmedetomidine, glycopyrrolate	4y	F	BV	N	cetirizine, ondansetron, cefazolin, propofol, glycopyrrolate, fentanyl, vecuronium
16y	M	BV	Y	levetiracetam, methylphenidate, lacosamide, trazodone, cefazolin, ketamine, glycopyrrolate, midazolam, propofol, rocuronium, fentanyl dexamethasone, hydromorphone	16y	F	PF	N	Cefazolin, propofol, fentanyl, morphine, ketamine, dexamethasone, toradol, acetaminophen	17y	M	BV	N	Protein supplement, MVI, cefazolin, propofol, fentanyl, ondansetron, glycopyrrolate, clonidine
12y	M	SC	N	risperidone, cimetidine, propofol, lidocaine, dexmedetomidine, dexamethasone, Benadryl, fentanyl, hydromorphone, ondansetron	15y	M	SC	N	ondansetron, midazolam, rocuronium, clindamycin, coenzyme Q10, levocarnitine, baclofen	11y	M	SC	N	cefuroxime, gentamycin, lorazepam, midazolam, dexamethasone
13y	F	BV	N	OCPs, midazolam, fentanyl, lidocaine, propofol, rocuronium, ondansetron	10y	F	PF	N	cefazolin, midazolam, propofol, dexamethasone, fentanyl	13y	F	PF	N	Cefazolin, propofol, dexmedetomidine, lidocaine, fentanyl, ketamine, ondansetron, midazolam
12y	F	BV	N	atomoxetine, darifenacin, clonidine, hydroxyzine, cefazolin, propofol, rocuronium, fentanyl, midazolam	10y	F	PF	Y	midazolam, cefuroxime, propofol, dexmedetomidine, fentanyl, ondansetron, morphine, dexamethasone	12y	F	PF	N	Cefazolin, propofol, rocuronium, ondansetron, midazolam, fentanyl, dexamethasone, dexmedetomidine
8y	F	BV	N	levetiracetam, loratadine, pyridoxine, leuprorelin, cefazolin, dexamethasone, propofol, rocuronium, fentanyl, midazolam, mannitol, dexmedetomidine	5y	F	BV	N	MVI, cefazolin, fentanyl, midazolam, dexmedetomidine, propofol	8y	F	PF	N	rocuronium, midazolam, mannitol, cefazolin

8y	M	BV	N	midazolam, vancomycin, cefuroxime, propofol, fentanyl, dexamethasone						8y	M	BC	N	Cefazolin, propofol, dexamethasone
4m	M	BV	N	Cefazolin, dexamethasone, ondansetron, atropine, fentanyl, midazolam	3m	M	BV	Y	levetiracetam, phenobarbital, propofol, rocuronium, fentanyl, glycopyrrolate, morphine, clonidine					
2y10m	M	SC	N	Cefazolin, propofol, dexmedetomidine, fentanyl dexamethasone, morphine, midazolam	3y	M	SC	N	Cefazolin, fentanyl, propofol, dexmedetomidine, ondansetron,	3y	M	SC	N	levetiracetam, ranitidine, cefuroxime, morphine, lidocaine, propofol, glycopyrrolate, Toradol, ketamine, dexmedetomidine
1y3m	M	SC	Y	cefazolin, propofol, rocuronium, fentanyl, ketamine, dexamethasone, ondansetron, glycopyrrolate	1y1m	M	SC	N	lansoprazole, cefazolin, fentanyl, midazolam, ondansetron, propofol	1y3m	M	SC	N	Cefazolin, fentanyl, dexamethasone, ondansetron, morphine
5y	M	BC	N	montelukast, fluticasone, salmeterol, fluticasone, cetirizine, cefazolin, propofol, fentanyl, lidocaine, ondansetron, dexmedetomidine						5y	M	BV	N	dexamethasone, vancomycin, pantoprazole, lorazepam, midazolam, fentanyl
9y	M	PF	N	Cefazolin,, midazolam, fentanyl, propofol, clonidine	9.5y	M	PF	N	Cefazolin, rocuronium, fentanyl, ketamine, dexmedetomidine, ondansetron, glycopyrrolate	9y	M	PF	N	triamcinolone, fluoxetine, cefuroxime, dexamethasone, ondansetron, propofol, fentanyl, dexmedetomidine
1m	M	BV	N	amoxicillin, gentamicin, vancomycin, TPN, propofol, fentanyl	1m	M	BV	N	furosemide, amiodarone, cefazolin, calciferol, lansoprazole, caffeine, acetaminophen, dexmedetomidine, TPN, fentanyl, glycopyrrolate,	3m	M	SC	N	Cefazolin, propofol, fentanyl, acetaminophen, dexmedetomidine
2y8m	M	BV	N	Cefazolin, propofol, rocuronium, fentanyl, ondansetron, cefazolin, glycopyrrolate, clonidine, midazolam						2y6m	M	PF	N	cefdinir, clindamycin, fentanyl, ondansetron, ketamine, dexmedetomidine, propofol, midazolam, rocuronium, glycopyrrolate
6y	M	SC	N	cetirizine, clonidine, Probiotic, melatonin, polyethylene glycol, cefazolin, dexmedetomidine, calcium gluconate, glycopyrrolate, fentanyl, midazolam,						6y	M	SC	N	polyethylene glycol, senna, cefazolin, fentanyl, ondansetron, dexamethasone, dexmedetomidine, propofol

8y	M	BC	Y	clonidine, dexamethasone, risperidone, levetiracetam, ondansetron						8y	M	BV	N	dexamethasone, propofol, morphine, fentanyl, cefuroxime, levetiracetam, melatonin, acetaminophen
18m	M	SC	N	Cefazolin, fentanyl, propofol, dexamethasone	2y	M	SC	N	Cefazolin, fentanyl, propofol, hydromorphone, dexmedetomidine, ondansetron, midazolam	1y6m	M	SC	N	polyethylene glycol, cefazolin, fentanyl, propofol, dexamethasone, clonidine, ketamine
3y	M	PF	N	cefuroxime, fentanyl, propofol, Ondansetron	4y	F	PF	N	cefuroxime, naproxen, clonidine, cyproheptadine, fentanyl, propofol, dexmedetomidine, glycopyrrolate, morphine, midazolam	3y	M	PF	N	Midazolam, dexamethasone, dexmedetomidine, fentanyl, morphine
4m	F	BV	Y	fentanyl, rocuronium, propofol, lidocaine, midazolam, neostigmine, glycopyrrolate, vancomycin, cefazolin, phenobarbital, levetiracetam, topiramate						5m	M	BC	Y	cefazolin, rocuronium, fentanyl, calcium, neostigmine, glycopyrrolate, phenylephrine, zonisamide, topiramate
12y	M	SC	Y	aripiprazole, clonidine, acetazolamide, lansoprazole, ibuprofen, guaifenesin, naproxen, pseudoephedrine, MVI, propofol, lidocaine, dexmedetomidine, cefazolin, ondansetron, morphine						12y	M	SC	N	Cefazolin, ketamine, rocuronium, fentanyl, dexamethasone, propofol, dexmedetomidine, ondansetron
12y	M	PF	N	oxcarbazepine, hydroxyzine, cefazolin, fentanyl, midazolam, propofol, vecuronium						12y	M	BV	N	Cefazolin, ondansetron, midazolam, fentanyl
10y	M	SC	N	Cefuroxime, dexmedetomidine, propofol, fentanyl, dexamethasone, hydromorphone						10y	M	SC	N	oxybutynin, cefuroxime, lidocaine, propofol, dexamethasone, dexmedetomidine, fentanyl, morphine
4y	M	BV	N	, mannitol, rocuronium, fentanyl, midazolam, heparin, dexmedetomidine, polyethylene glycol, acetaminophen, morphine, cefazolin	4y	M	BC	Y	levetiracetam, clonazepam, cefazolin, fentanyl, propofol, dexamethasone, ondansetron, dexmedetomidine	4y	M	PF	N	Cefazolin, propofol, dexmedetomidine, fentanyl, midazolam, ondansetron
3m	M	BV	N	cefazolin, rocuronium, fentanyl						6m	M	BV	N	clonidine, lansoprazole, cefuroxime,

														dexmedetomidine, propofol, fentanyl, dexamethasone
6y	M	PF	Y	atomoxetine, fluticasone, albuterol, cetirizine, iron, MVI, Propofol, fentanyl, dexamethasone, ondansetron, dexmedetomidine	6y	M	PF	N	midazolam, cefazolin, fentanyl, rocuronium, propofol, dexmedetomidine	6y	M	PF	N	Cefuroxime, fentanyl, rocuronium, ondansetron, propofol, dexmedetomidine, metoclopramide
6y	M	SC	N	polyethylene glycol, cefazolin, propofol, fentanyl, dexamethasone, ondansetron						6y	F	SC	N	Cefazolin, fentanyl, ondansetron, propofol, Toradol, dexmedetomidine, acetaminophen, morphine
15y	M	BC	N	Cefazolin, propofol, lidocaine, rocuronium, glycopyrrolate, hydralazine, midazolam, fentanyl, dexmedetomidine	8y	M	BV	N	budesonide, cefazolin, fentanyl, albuterol, lidocaine, propofol, rocuronium, ondansetron, dexamethasone, ketamine, dexmedetomidine	15y	M	BV	N	ondansetron, Morphine, dexamethasone, pantoprazole, cefazolin
9y	M	BC	Y	oxcarbazepine, lamotrigine, atomoxetine, guanfacine, clonazepam, cefazolin lidocaine, propofol, dexamethasone, ondansetron, rocuronium, midazolam, glycopyrrolate, dexmedetomidine, morphine						9y	M	PF	N	Cefuroxime, midazolam, propofol, dexmedetomidine, fentanyl, lidocaine, dexamethasone, ondansetron hydromorphone
6y	M	BC	Y	fentanyl, rocuronium, ketamine, propofol, promethazine, acetaminophen, phenylephrine, dexmedetomidine, morphine, oxcarbazepine clonazepam, albuterol, Pulmicort, clobazam, guanfacine, CBD						6y	M	PF	N	dexamethasone, vancomycin, fentanyl, rocuronium, mannitol, glycopyrrolate, propofol, midazolam, dexmedetomidine, dopamine
6y	M	SC	N	aspirin, enalapril, sildenafil, furosemide, lansoprazole, ciprodex, cefazolin, fentanyl, midazolam, dexmethasone, rocuronium, dexmeditomidine, propofol						6y	M	SC	N	midazolam, cefazolin, propofol, dexamethasone, fentanyl, dexmedetomidine
4y	F	BS	N	cefazolin, propofol, fentanyl, midazolam, dexamethasone, ondansetron, albuterol, fluticasone, polyethylene glycol, simethicone, fexofenadine, diphenhydramine	4y	F	PF	N	fluticasone, albuterol, cefuroxime, propofol, fentanyl, dexmedetomidine, ondansetron, morphine	4y	F	BV	N	vancomycin, cefepime, fentanyl, propofol, ondansetron, suxamethonium, cefazolin, dexamethasone, amoxicillin

8y	M	SC	Y	oxybutynin, albuterol, cefazolin, dexmedetomidine, midazolam, fentanyl	7y6m	M	SC	N	Cefazolin, dexmedetomidine, dexamethasone, fentanyl	8y	M	SC	N	Cefazolin, propofol, dexamethasone, hydromorphone, ondansetron
12y	M	BC	Y	Cefazolin, fentanyl, epinephrine, vasopressin, norepinephrine, midazolam						12y	M	PF	N	amitriptyline, Imitrex, naproxen, albuterol, cefuroxime, fentanyl, lidocaine, propofol, dexamethasone, ondansetron, dexmedetomidine, midazolam
9y	M	BC	Y	Cefazolin, ketamine, fentanyl, morphine, ondansetron, rocuronium, propofol, propranolol, promethazine, dexmedetomidine						9y	M	PF	N	Cefazolin, propofol, rocuronium, fentanyl, ondansetron, dexmedetomidine, midazolam
5y	M	BC	Y	Cefazolin, ketamine fentanyl, propofol, ondansetron, rocuronium	5y	F	PF	Y	levetiracetam, cefazolin, fentanyl, dexmedetomidine, dexamethasone, morphine sulfate, midazolam, albuterol					

Abbreviation: BC: Brain Cortex; BS: Brain Stem; BV: Brain Ventricle; PF: Brain Posterior Fossa; MVI: multivitamin; OCP: Oral Contraception Pills; SC: Spinal Cord; TPN: Total Parental Nutrition
